# The Air We Breathe: Effect of Environmental Exposures on COPD

**Published:** 2017

**Authors:** Jennifer Quint

**Affiliations:** National Heart and Lung Institute, Imperial College London

Chronic obstructive pulmonary disease (COPD) is a chronic progressive disease associated with the abnormal inflammatory response of the lungs to noxious particles or gases. The inflammatory responses can lead to increased sputum production, breathlessness and reduced lung function, often resulting in reduced exercise tolerance and decreased quality of life. Currently COPD is the 4th leading cause of death worldwide and it is predicted that deaths from COPD may increase by more than 30% in the next 10 years.

While smoking is the most important risk factor for COPD, approximately 25–45% of patients have never smoked. The prevalence of COPD is higher in individuals living close to traffic and COPD patients have substantial mortality risks associated with particles and temperature changes.

Previous systematic reviews have focused on the association between COPD exacerbations with exposure to particulate matter. One study, estimated a 2.7% increase for COPD hospital admissions (95% CI: 1.9%–3.6%) for every 10 μg/m^3^ increase in PM_10_, and reported large heterogeneity in effect estimates from *I^2^* = 83.9% to 79.4% (13). In a recent systematic review we undertook, we found a marginally significant effect estimate for PM_10_ (1·01, 95% CI: 1.0–1.02). Song found that the strength of the association of COPD hospital admissions with PM_10_ varied among geographical locations with an effect of 1% in China and Europe but a larger effect of 2% in the United States. We estimated a similar effect of 1% in Europe with very little heterogeneity (*I^2^*=1.93%); however a smaller effect of 1% in North America, and a larger effect in Asia of 3% (95% CI: 2% to 5%). Only one meta-analysis estimated the association of COPD admissions (excluding asthma) with PM_2·5_ exposure and found a similar association 1.02 (95% CI: 1.01–3.71) to our study (OR: 1.03, 95% CI: 1.01–1.05). Large multicity studies in North America and Europe failed to detect a significant association between outdoor PM_10_ levels and COPD hospital admissions. Two multicity studies in North America did not find a significant association between PM_2·5_ exposure and COPD hospital admissions. The effect of seasonal variation on the association between PM_10_ exposure and COPD exacerbations is not clear.

The association between COPD exacerbations with gaseous pollutants indicates a potential link between CO and SO_2_ levels with moderate heterogeneity and strong geographical clustering. The effect estimates of SO_2_ in each geographical subgroup indicated that the association was only significant in Asian countries with stronger effect in the winter season, marginally significant in Europe, and insignificant in North America where the majority evidence comes from, possibly because SO_2_ remains a predominant pollutant in developing countries. The only two available multi-city studies on the effects of SO_2_ found contradictive results; one study in Europe reporting a marginal positive association and one study in North America reported a negative non-significant association. The associations between NO_2_ and O_3_ exposure with COPD hospital admissions is less well understood.

What is clear, is that the effect of separate pollutants on COPD admissions appears to vary across geographical regions. Effects are evident even at concentration below current guideline values indicating the need to lower thresholds to protect such vulnerable groups. Of course what is seen at ecological level is not always true at a personal level in terms of exposure and there has been a continued effort to understand the relationship between ambient concentrations and personal exposure. Personal exposure assessment requires the recording of a person’s time-activity patterns, as well as the pollutant concentrations which each individual is exposed to. At the most basic level, this may be the relative proportion of time spent in different microenvironments. Additionally, activity type of individuals may affect indoor air pollution levels, while activity levels may alter dose.

Our current research project takes the first steps towards the integration of novel methodological approaches in three main areas : 1) the recruitment of participants via an anonymised GP records database, and use of primary care electronic records to gather information on COPD exacerbations; 2) mass deployment of portable air quality sensor platforms over long periods revolutionising the way in which personal exposure can be quantified with automated classification of individual time-activity patterns and exposure events; and, 3) the application of a dynamic human exposure model. Together, these have the potential to provide powerful tools to create and validate accurate personal exposure models with higher spatio-temporal resolution, allowing, for the first time, the incorporation of spatially realistic exposure models in epidemiological studies.

The strength of this study is in the fact we will have the ability to assess these associations in far more detail, initially at individual patient level and potentially at a national level in the UK. For the first time, this study will provide a multi-disciplinary methodological framework that will bring together recent advancements in low-cost wearable sensors, computational techniques for the estimation of activity-weighted personal exposure and advanced spatial mapping in a well characterised population study. The integrated database of environmental stressors and activity patterns at the individual level will form the basis for the validation of the human exposure London model.

Ultimately we will aim to develop forecasting models that can be used to predict times of increased exacerbation risk. This will aid health care providers and allow more accurate planning and allocation of resources which will reduce costs for the NHS. It will aid patients as it may provide an opportunity to alter behaviour and to prevent exacerbations from occurring. By providing a more robust evidence base, policy makers may be able to take more targeted and efficient decisions on reducing environmental risk. Members of the public will be able to make more informed decisions on how to minimise their own risks, improving health and quality of life.

